# Thoracic vertebral metastasis from progressive hepatocellular carcinoma following liver transplantation combined with resection of mesenteric and colonic metastases: A case report

**DOI:** 10.1097/MD.0000000000022937

**Published:** 2020-10-30

**Authors:** Jingen Hu, Caibao Hu

**Affiliations:** a Department of Orthopedics, The First Affiliated Hospital, School of Medicine, Zhejiang University; b Intensive Care Unit, Zhejiang Hospital, Hangzhou, Zhejiang, China.

**Keywords:** En Bloc spondylectomy, hepatocellular carcinoma, liver, spinal metastasis, transplantation

## Abstract

**Rationale:**

Surgical treatment of spinal hepatocellular carcinoma metastasis after Liver transplantation (LT) is a clinical challenge. We herein report the clinical outcomes of the first case of a patient with T11 from hepatocellular carcinoma metastasis after systemic chemotherapy following LT combined with mesenteric resection and colectomy, who was successfully treated with En Bloc spondylectomy.

**Patient concerns:**

The patient with HCC was a 40-year-old man, who had received LT combined with mesenteric resection and colectomy 15 months before. His main symptom was progressive back pain because of T11 metastasis. PET examinations showed a solitary metastasis at T11 without recurrence in the liver and metastasis in the other organs.

**Diagnosis:**

The patient was diagnosed with the T11 vertebra HCC metastasis after LT combined with resection of HCC mesenteric metastasis and colon metastasis.

**Interventions:**

Five cycles of systemic chemotherapy following LT were performed for preventing HCC metastases. However, the right abdominal wall metastasis was found 9 months after LT, followed by T11 metastases thereafter. Immediate resection of the right abdominal wall metastasis was achieved. En Bloc spondylectomy of T11 vertebra was chosen as a treatment for metastasis to T11. After T11 surgery, the patient showed obvious pain relief. However, At 3 months after T11 surgery, a grafted liver metastasis and multiple nodules metastasis in the greater omentum region were revealed with CT imaging, At 5 months after T11 surgery, multiple lung metastases were discovered by MRI. The patient was performed 5 cycles of chemotherapy, 3 times of infusion of iodine [131I] meximab and 3 times of TACE after T11 surgery. Multiple bone metastases were treated with radiotherapy.

**Outcomes:**

The patient died 29 months after LT combined with mesenteric resection and colectomy because of recurrence in the liver and metastasis in the lung.

**Lessons:**

En Bloc spondylectomy may be a therapeutic choice for patients with progression after systemic chemotherapy for the solitary spinal metastases after LT combined with mesenteric resection and colectomy, which has a survival benefit without local recurrence at the surgical site. immunosuppressant after LT may result in worse immune function, which leads to HCC more prone to recurrence and bone metastasis.

## 1 Introduction

Approximately 20% of bone metastases (BMs) are caused by extrahepatic metastasis.^[1]^ The onset of BMs secondary to hepatocellular carcinoma (HCC) has been relatively rare compared with other types of cancer but has recently increased. In the past, the presence of BMs associated with HCC was considered rare and the outcomes were typically poor.^[2]^ However, with recent advances in radiographic imaging methods and treatment modalities, BMs in patients with HCC are more commonly detected. Liver transplantation (LT) is the curative therapy for early HCC, but LT for HCC maintains a risk of HCC metastasis and recurrence after LT.^[3]^ Few reports are available exploring operative approaches for bone metastases after LT secondary to HCC. In this report, we present our experience of operative approaches for bone metastases and recurrence after LT combined with mesentery and colectomy management in this case.

## 2 Case report

**Figure 1:**
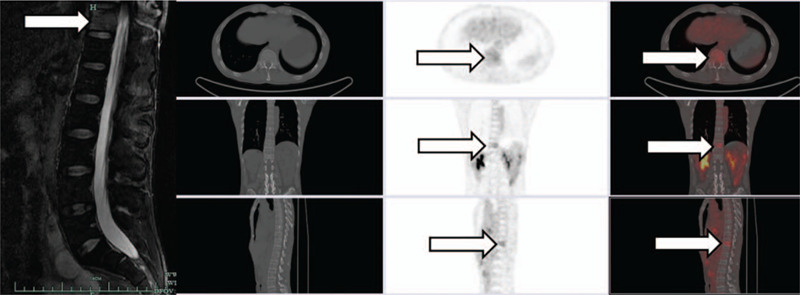
Preoperative radiographic images. Sagittal views of the T1-weighted magnetic resonance images (left) and PET (right) images. Arrows indicate the T11 metastasis 15 months after LT.

**Figure 2:**
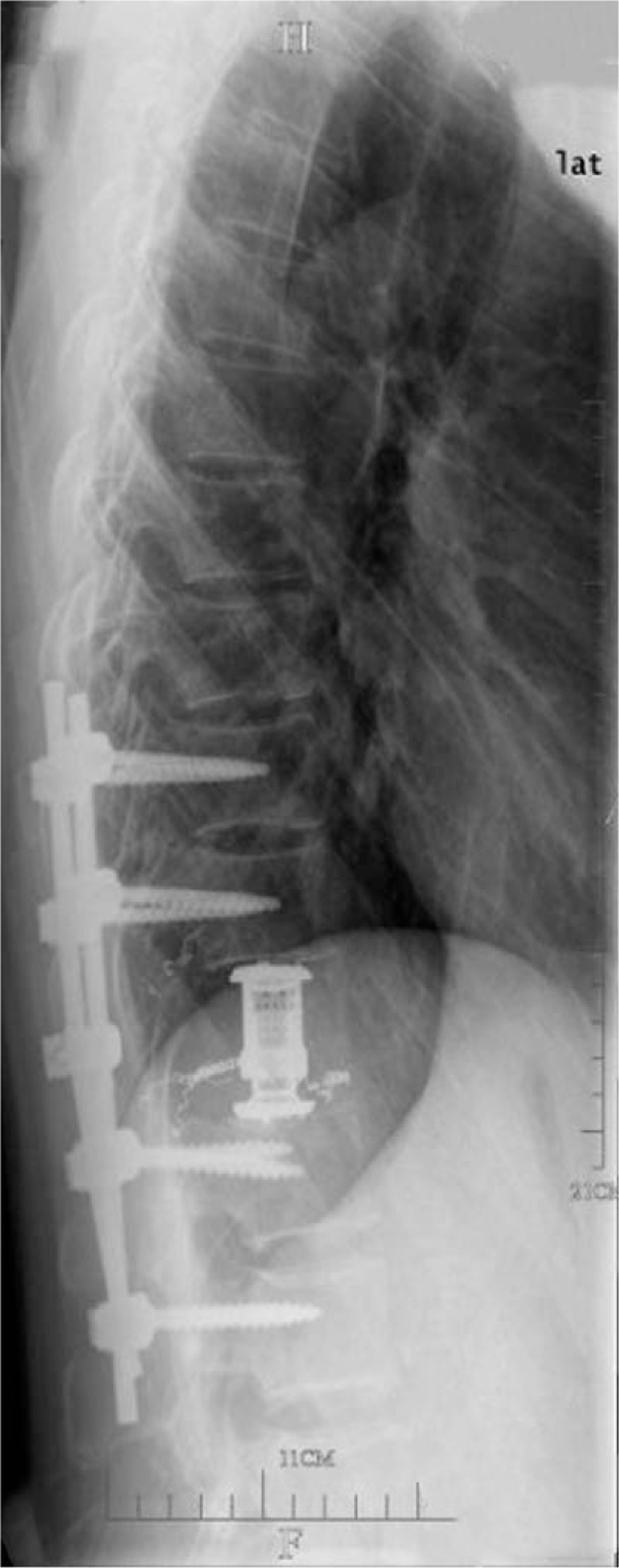
Lateral view of radiographs after total en bloc spondylectomy for T11 metastasis.

A 40-year-old man, who had undergone LT combined with partial resection of the small-bowel mesentery and transverse colon for HCC 15 months before, complained of back pain for 1 month. The patient underwent 5 cycles of chemotherapy within 7 months after LT and resection of the right abdominal wall metastasis 9 months after LT. A solitary bone metastasis was found by MRI and PET (Fig. 1) in T11 vertebra 15 months after LT, which did not cause pathological fracture or spinal cord compression. No new metastatic tumors were found in the previously resected mesentery, colon and right abdominal wall, and no metastasis to the grafted liver or other major internal organs were found, though the T11 lesion was found, indicating progressive disease after chemotherapy. The patient's Tomita score and modified Tokuhashi score for evaluating the prognosis of patients with spinal metastatic tumors were 5 and 10 points, respectively.^[4,5]^ The patient underwent an En Bloc spondylectomy of T11 vertebra metastases using a posterior approach. The pedicle screws were inserted into both sides of the T9, T10, T12, and L1 vertebrae through the pedicle, and then the titanium alloy rods were fixed on the screws. Reconstruction was achieved by using a artificial vertebral body at the original T11 position after the T11 En Bloc spondylectomy (Fig. 2). No complications occurred. A histological analysis of the T11 vertebra after surgery revealed HCC metastasis (moderate-poorly differentiated HCC).

**Figure 3:**
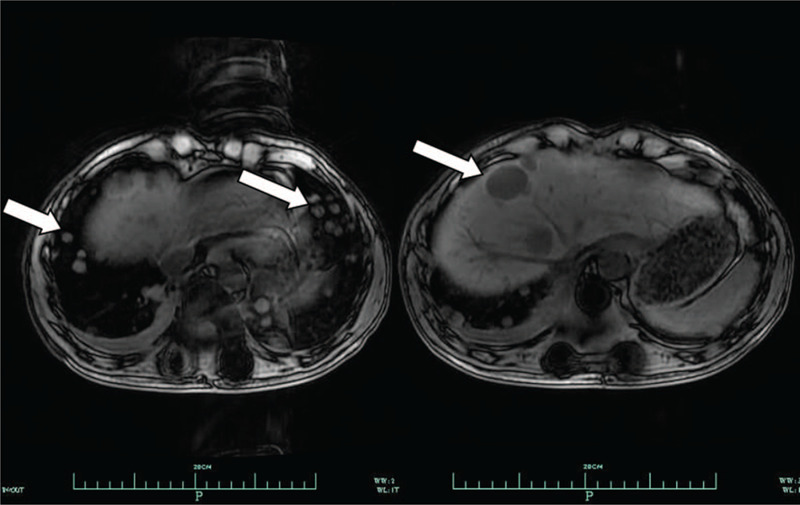
MRI imaging indicated metastasis at the grafted liver and lung 5 months after en bloc spondylectomyof T11. Arrows indicate recurrence of hepatocellular carcinoma of grafted liver and the newly developed metastasis at lung.

After T11 surgery, his AFP levels decreased markedly. However, his AFP levels increased rapidly again 3 months after T11 surgery, and MRI imaging indicated metastasis at the grafted liver and the greater omentum. Numerous lung metastases were discovered by MRI (Fig. 3) 5 months after en bloc spondylectomy of T11. The patient was performed 5 cycles of chemotherapy, 3 times of iodine [131I] rituximab infusion and 3 times of TACE after T11 surgery. Radiotherapy was performed for multiple lung metastases to alleviate clinical symptoms. After radiotherapy, the pain symptoms of the patient were significantly improved, followed by numerous bone metastases thereafter, but no tumor recurrence occurred at the T11 surgical site. Radiotherapy was also used to treat multiple bone metastases. He ultimately died of recurrence in the grafted liver and metastasis in the lung 29 months after LT.

## 3 Discussion

To determine the most appropriate treatment, various scoring systems, including Tomita, Katagiri, and Modified Tokuhashi scores, are used to predict survival and post-treatment outcomes in patients with spinal metastases.^[4–6]^ However, many of these scoring systems may be inappropriate because they have not yet been adjusted or improved to reflect some of the latest advances in cancer treatment, that is, organ transplants, TACE, radiotherapy, and targeting drugs. These advances have had a profound impact on the survival of patients with spinal metastasis. In addition, these scoring systems do not include prognostic scores for patients undergoing LT combined with resection of other internal organs from HCC.

Previous studies by our team have shown that the median survival time after bone metastasis (BM) in LT (16 cases) and non-LT patients (27 cases) was 20 and 11 months, respectively. This did not mean that the median survival time of LT patients is longer than that of non-LT patients.^[7]^ On the contrary, these results reflect the nature of the patient population. Compared with non-LT patients, LT patients are typically diagnosed as early HCC, with better intrahepatic tumor control rather than extending to soft tissue.^[7]^

The majority of patients with recurrences after LT have multiple sites of disease spanning different therapeutic fields. Surgeries for spinal metastases are effective for removing masses, while decompression with resection helps to improve pain and alleviate neurological blockade. The walking condition of patients coupled with radiation therapy after surgery was significantly improved compared with patients undergoing radiation alone despite some reports contradicting that with the results a study conducted by Rades and colleagues.^[8]^ The operative procedure is considered to be a factor determining survival,^[9]^ so patients who develop a recurrence after transplantation have higher life expectancy if they undergo surgery.

Studies have shown that in patients receiving LT for HCC,^[9–12]^ bone is the third largest extrahepatic metastasis site after grafted liver and lung (18–33%). Subsequent studies identified several clinical characteristics and prognostic factors of bone metastasis after LT for HCC. The most frequently affected site in these patients was the thoracic vertebrae, accounting for 15.1% of all patients.^[13]^ The median survival time in this group was 8.6 months, and liver failure or tumor progression was the most common cause of death. In our case, the patient was found to have metastasized to the grafted liver, lung, and bone. Despite the active treatment of spinal metastasis, additional metastases on the grafted liver and lungs still exist, resulting in progressive decompensation of liver function and respiratory failure caused by lung metastasis. The patient eventually died of the disease 29 months after LT. Among the affected sites, the bone metastasis responded poorly to irradiation, with the bone lesion continuing to grow larger and more over time.

The patient developed bone metastasis 15 months after systemic chemotherapy following LT combined with mesenteric resection and colectomy, suggesting that the patient has two possibilities. The first possibility is bone metastasis during LT, which was simply unable to be detected by current diagnostic methods. The second case is that immunosuppression after LT leads to immune insufficiency, which will make HCC more prone to recurrence and bone metastasis. In this study, we prefer the latter interpretation because the first bone metastasis in our patient was found after 15 months of immunosuppression therapy following LT rather than immediately after LT.

The patient's burden of disease including the immune status was early detected and assessed by the LT team from the time of transplantation in our hospital. Studies have shown that post-transplant recurrence is more severe than that post-hepatectomy because the immunosuppressive drugs used after transplantation have many side effects on the overall immunity of the patient.^[1]^ Patients with LT usually receive immunosuppressive therapy after LT,^[14,15]^ which can accelerate tumor recurrence and metastasis. At present, there is no reliable conclusion that any specific immunosuppressants have an effect on the recurrence of HCC; however, compared with other factors, immunosuppressive load seems to play a decisive role in the recurrence of HCC.^[16,17]^ The original immune state of patients can increase the survival rate of patient and graft,^[18–20]^ the current trend is to take into account the original immune state of patient, as far as possible to reduce the immunosuppressive load. This type of treatment usually requires appropriate adjuvant chemotherapy to minimize immunosuppression.^[21]^

When long-term survival is expected, posterior decompression for symptomatic spinal metastasis is very common, as well as total En Bloc spondylectomy for a single metastatic site.^[4]^

Several studies have reported that En Bloc spondylectomy has a survival benefit in patients with solitary spinal metastasis derived from different tumors.^[22–25]^ When we review the literature, only two case reports^[26,27]^ have investigated the application of En Bloc spondylectomy in the treatment of HCC solitary spinal metastasis after LT.

Hu et al^[26]^ reported that the preceding radiotherapy along for solitary T12 vertebra metastasis after LT in a case report was unable to control tumor progression. Accordingly, the patient was treated via En Bloc spondylectomy of T12. After surgery of T12, the patient developed an infection of the spinal incision, which was thought to be related to radiotherapy. Based on the patient's medical condition, the patient was predicted to have a very short survival and a poor overall quality of life; however, the patient ultimately lived beyond the predicted survival time of 6- to 12-month and no recurrence at the T12 surgical site was noted at a follow-up postoperatively.

Hiroaki et al^[27]^ reported that a case underwent radiation therapy for solitary T7 vertebra metastases after LT, but the paralysis in the lower legs aggravated further, which was thought to have been the result of spinal cord injury caused by radiotherapy. Accordingly, the patient was treated via En Bloc spondylectomy of T7 through a posterior approach. The patient was satisfied with the treatment without sign of recurrence at the T7 site of the operation at the 1.5-year follow-up.

From these only two case reports concerning En Bloc spondylectomy for solitary spinal metastasis from HCC after LT, two patients all received radiotherapy first, but radiotherapy could not control tumor progression^[26]^ and injure the spinal cord,^[27]^ and then they was treated via En Bloc spondylectomy for solitary spinal metastasis from HCC after LT.

We also considered radiotherapy before En Bloc spondylectomy for solitary T11 metastasis after LT, but radiotherapy has the risk of losing control of tumor development^[26]^ and damaging spinal cord,^[27]^ while En Bloc spondylectomy can theoretically completely remove the tumor and reduce the risk of local recurrence. This patient had appropriate liver function without HCC recurrence in the right abdominal wall after resection of right abdominal wall metastasis following LT and no metastases except the solitary T11 metastasis. Therefore, we performed En Bloc spondylectomy, and expected for long-term survival and long-term local control. The patient ultimately survived beyond the predicted time of 6- to 12-month and no recurrence at the T11 surgical site was seen at the 14-month follow-up postoperatively.

The case in our study showed En Bloc spondylectomy had a survival benefit without local recurrence at the T11 surgical site in the treatment of HCC solitary spinal metastasis after LT combined with mesenteric resection and colectomy, which is consistent with the results of previous only two case reports^[26,27]^ concerning En Bloc spondylectomy for solitary spinal metastasis from HCC after LT.

Compared with only receiving LT patients in previous only two case reports,^[26,27]^ our patient receiving LT combined with mesenteric resection and colectomy were typically diagnosed as advanced HCC, with poorly controlled intrahepatic tumors that extend to surrounding mesenteric and colonic soft tissue. In our patient due to the mesentery and colon invasion by the tumor, a mesenteric resection and colectomy was performed during LT. When we review the literature, we could not find any other HCC in liver case with a mesentery and colon invasion which was treated by LT combined with mesenteric resection and colectomy. We did not preserve the mesentery and colon due to it being full of metastasis in the mesentery and colon. It is unclear yet, whether a mesentery and colon preserving surgery of LT is suitable or safe for this visceral organs HCC metastasis.

Taking into consideration that the prediction of patients with HCC with spinal metastasis is poor, a healthy liver and good liver function after LT may enable a successful surgery.^[27]^ The patient here had good coagulopathy and liver function without surgical contraindications at the time of discovery of the bony metastases and the first bone metastasis surgery.

To the best of our knowledge, no reports have been published about En Bloc spondylectomy for solitary spinal metastasis of HCC after LT combined with surgery of other visceral organs for HCC. Based on our experience, we suggest that only patients with solitary spinal metastases and appropriate liver function after LT combined with surgery for metastasis of other visceral organs consider any surgical intervention, which is consistent with the results of previous researchers.^[28]^ The case presented here suggests that total En Bloc spondylectomy may be a feasible treatment option for patients with solitary spinal metastases after LT combined with mesenteric resection and colectomy, which had a survival benefit without the surgical site recurrence.

## Author contributions

**Conceptualization:** Jingen Hu, Caibao Hu.

**Data curation:** Jingen Hu.

**Formal analysis:** Jingen Hu.

**Investigation:** Jingen Hu.

**Resources:** Jingen Hu.

**Supervision:** Jingen Hu.

**Validation:** Jingen Hu.

**Writing – original draft:** Jingen Hu.

**Writing – review & editing:** Caibao Hu.
